# 499. Rapid and Sustained Decline in CXCL-10 (IP-10) Annotates Clinical Outcomes Following TNF-α Antagonist Therapy in Hospitalized Patients with Severe and Critical COVID-19 Respiratory Failure

**DOI:** 10.1093/ofid/ofab466.698

**Published:** 2021-12-04

**Authors:** Courtney Schroeder, Hilal Hachem, Amandeep Godara, Daniel Fein, Hashim Mann, Christian Lawlor, Jill Marshall, Andreas Klein, Debra Poutsiaka, Janis Breeze, Raghav Joshi, Paul Mathew

**Affiliations:** 1 Tufts Medical Center, Boston, Massachusetts; 2 Eastern Maine Medical Center, Bangor, Maine; 3 University of Utah School of Medicine, Salt Lake City, Utah; 4 Tufts Medical center, Boston, Massachusetts

## Abstract

**Background:**

TNFα and IFN-γ may synergize to induce cytokine-driven lethal hyperinflammation and immune exhaustion in COVID-19 illness.

**Methods:**

To assess TNFα-antagonist therapy, 18 hospitalized adults with hypoxic respiratory failure and COVID-19 pneumonia received single-dose infliximab-abda therapy 5mg/kg intravenously between April and December 2020. The primary endpoint was time to increase in oxygen saturation to fraction of inspired oxygen ratio (SpO2/FiO2) by ≥ 50 compared to baseline and sustained for 48 hours. Secondary endpoints included 28-day mortality, dynamic cytokine profiles (Human Cytokine 48-Plex Discovery Assay), secondary infections, duration of supplemental oxygen support and hospitalization.

Consort diagram

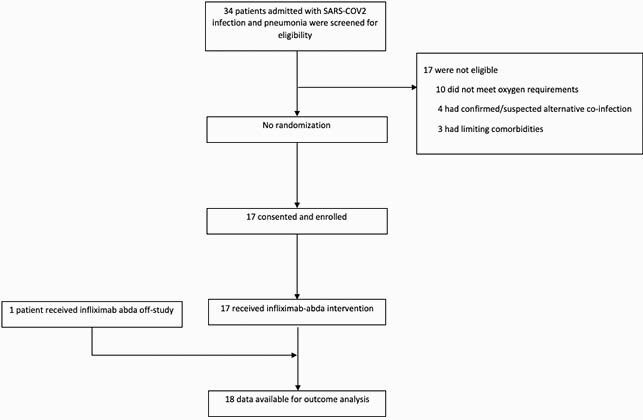

Hospitalized patients with SARS-COV2 infection and pneumonia that were referred to the infliximab-abda study team for evaluation.

**Results:**

Patients were predominantly in critical respiratory failure (15/18, 83%), male (14/18, 78%), above 60 years (median 63 yrs, range 31-80), race-ethnic minorities (13/18, 72%), lymphopenic (13/18, 72%), steroid-treated (17/18, 94%), with a median ferritin of 1953ng/ml. Sixteen patients (89%) met the primary endpoint within a median of 4 days, 15/18 (83%) recovered from respiratory failure, and 14/18 (78%) were discharged in a median of 8 days and were alive at 28-day follow-up. Deaths among three patients ≥ 65 years age with pre-existing lung disease or multiple comorbidities were attributed to secondary lung infections. Mean plasma IP-10 levels declined sharply from 9183 pg/ml to 483 pg/ml at Day 3 and 146 pg/ml at Day 14/discharge. Significant declines in IFN-γ, TNFα, IL-27, IL-6 (baseline above 10pg/ml), CRP and ferritin were specifically observed at Day 3 whereas other cytokines were unaffected. Among 13 lymphopenic patients, six (46%) had resolution of lymphopenia by day 3, and 11 by day 14. CXCR3-ligand (IP-10 and CXCL-9) declines were strongly correlated among patients with lymphopenia reversal (Day 3, Pearson r: 0.98, p-value: 0.0006).

Demographics and clinical characteristics

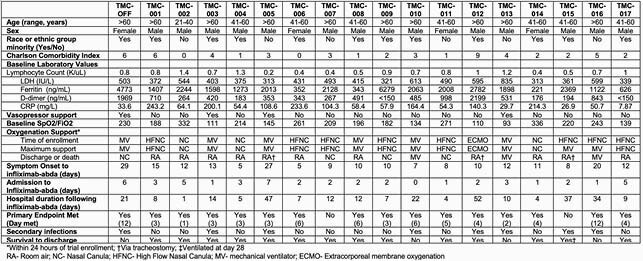

Demographics, comorbidities, clinical features, inflammatory markers, and outcomes of 18 patients with COVID-19 respiratory failure treated with infliximab-abda between April and December 2020.

Changes in oxygen support status following infliximab-abda treatment

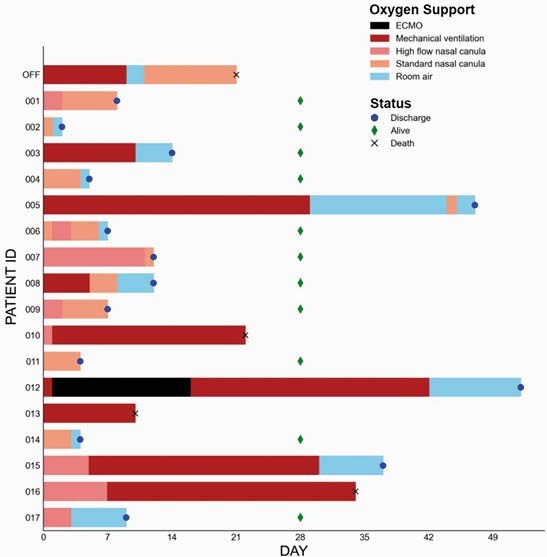

Colored bars indicate the maximal level of oxygen support for each individual following treatment with infliximab-abda. The status of the patient at last follow-up (discharged, alive or dead) is indicated. ECMO: extracorporeal membrane oxygenation

Control of inflammatory markers and cytokines following infliximab therapy

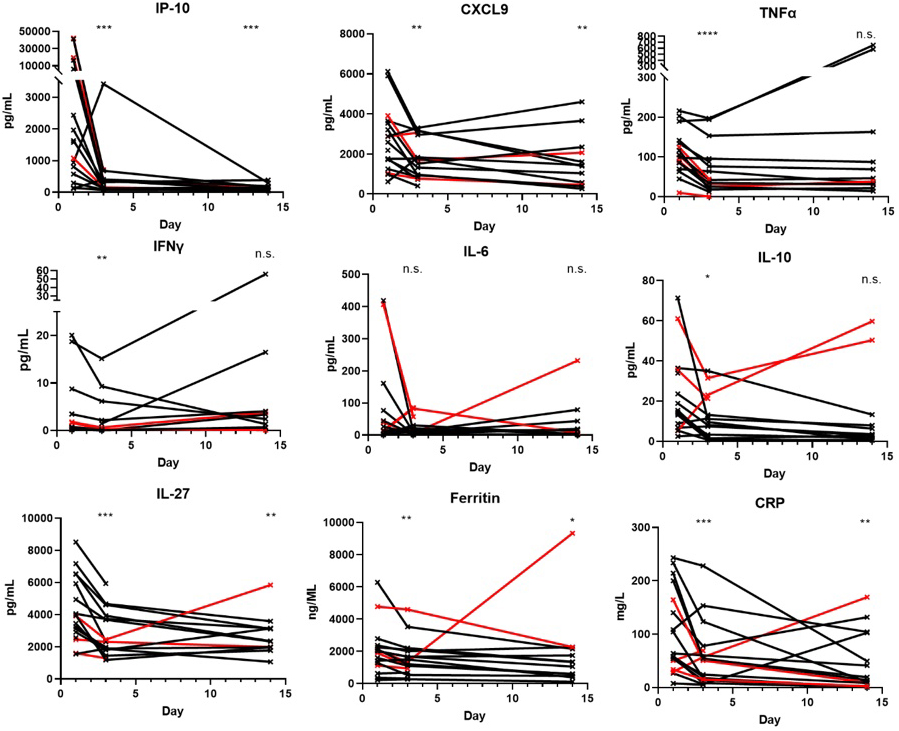

Values from individuals are connected with solid lines, with deceased individuals indicated in red. Statistics: n=18, paired ratio t-test compared to baseline; *: P<0.05, **: P<0.01, ***: P<0.001, ****: P<0.0001, n.s.: not significant.

**Conclusion:**

Consistent with a central role of TNFα, the clinical and cytokine data indicate that infliximab-abda may rapidly abrogate pathological inflammatory signaling to facilitate clinical recovery in severe and critical COVID-19. Randomized studies are formally evaluating infliximab therapy in this context. Funding: National Center for Advancing Translational Sciences

**Disclosures:**

**All Authors**: No reported disclosures

